# Great gerbil burrowing-induced microbial diversity shapes the rhizosphere soil microenvironments of *Haloxylon ammodendron* in temperate deserts

**DOI:** 10.3389/fmicb.2022.960594

**Published:** 2022-08-10

**Authors:** Hanli Dang, Wenqin Zhao, Tao Zhang, Yongxiang Cheng, Jianrui Dong, Li Zhuang

**Affiliations:** ^1^College of Life Sciences, Shihezi University, Shihezi, Xinjiang, China; ^2^Xinjiang Production and Construction Corps Key Laboratory of Oasis Town and Mountain-basin System Ecology, College of life Sciences, Shihezi University, Shihezi, Xinjiang, China; ^3^Key Laboratory of Oasis Eco-agriculture, College of Agriculture, Shihezi University, Shihezi, Xinjiang, China

**Keywords:** desert ecosystem, gerbil disturbance, *haloxylon ammodendron*, microbial diversity, soil microhabitat

## Abstract

In the Gurbantunggut Desert of northwest China, the main habitat of *Rhombomys opimus* (great gerbil) is under the thickets of *Haloxylon ammodendron*, the main construction species. In the long-term coexistence, continuous gerbil activities (burrowing, defecating, and gnawing) limited the growth of *H. ammodendron*, affected the root microenvironment under the *H. ammodendron* forest, and weakened the desert ecosystem. However, there is a lack of general understanding about the response of desert soil microhabitats to such gerbil disturbance. Accordingly, this study examined the effects of different intensities of gerbil disturbance (none, mild, moderate, or severe disturbances) on soil nutrients content and used high-throughput sequencing to explore the change in diversity and structure of microbial communities (bacteria and fungi) in *H. ammodendron* rhizosphere at different soil depths (0–20, 20–40, and 40–60 cm). In the arid desert ecosystem, compared with the soil fungal community, the alpha diversity of the soil bacterial community was significantly affected by gerbil disturbance. Meanwhile, both soil depth and gerbil disturbance significantly impacted the beta diversity and relative abundance of soil bacterial and fungal communities. In addition, gerbil disturbance significantly altered the soil characteristics affecting the distribution and composition of soil microbial communities in *H. ammodendron* rhizosphere, especially the soil bacterial community. This survey provides evidence that remold impact of gerbil disturbance on soil microenvironment of *H. ammodendron* rhizosphere in desert ecosystems in northwest China, which helps to further understand the potential correlations with changes in the microbial community at a regional scale.

## Introduction

The patterns of ecological community diversity and spatial composition reflect their inherent adaptability and flexibility to external environmental conditions including physical disturbances (Wittebolle et al., 2009). Notably, the disturbance from rodent activities on soil and vegetation has attracted the attention worldwide. Desert rodents act as ecosystem engineer in different ecosystems through burrow structure, excretion behavior, dietary composition, and foraging behavior (Lin et al., 2010; Kuznetsova et al., 2013). The phenomenon can be attributed to the influence of burrowing behavior on soil aeration, moisture, nutrient status, structure, and microtopography (Reichman and Seabloom, 2002; Kuznetsova et al., 2013), thus affecting vegetation productivity and community dynamics. Such spatial changes in species diversity and composition often lead to functional changes impacting important ecosystem processes, including productivity, stability, nutrient cycling process, and resistance to interference (Petchey and Gaston, 2002; Cadotte et al., 2011). Therefore, elucidating the effects of rodent disturbance on soil microhabitats is essential to understand their impact on the balance of an ecosystem, especially the desert ecosystem with relatively poor soil nutrients.

One challenge in ecological researches is to understand the assembly patterns and associated assembly processes of biomes in response to environmental changes (Dini-Andreote et al., 2015; Guo et al., 2020), including the uncertain impact of rodent activity. In desert ecosystems, rodents’ urine and feces can increase soil nutrient availability promoting soil microbial metabolic activity (Lin et al., 2012), which in turn impacts the composition and structure of the soil microbial community (Kuznetsova et al., 2013). Soil microorganisms significantly contribute to the biogeochemical cycle of major elements (carbon, nitrogen, and phosphorus), decomposition of organic matter (e.g., animal carcasses and leaf litter), and soil structure (Bach et al., 2010; Tripathi et al., 2018; Ren et al., 2021). For these reasons, the assembly of soil microbial communities has become a prime focus in microbial ecology studies. Studies have shown that the activities of rodents, such as *Cynomys ludovicianus* and *Dipodomys spectabilis* contribute to landscape heterogeneity, soil resources spatial heterogeneity, and the richness of plant species in the desert ecosystem (Ceballos et al., 1999; Ayarbe and Kieft, 2000), which directly or indirectly influences the structure and function of soil microbial community. In general, the structure and function of microbial communities change along the environmental/experimental gradient (Marschner et al., 2003; Fierer et al., 2012). The taxonomic structure of microbial communities is more sensitive to changes in environmental conditions than to their contribution to ecosystem function. Although environmental disturbance can significantly change the richness of microbial composition, microbes can still play a functional role in the ecosystem due to a high degree of functional redundancy (Berga et al., 2012). However, until now, it is not clear how microbial-related taxa respond to animal disturbance affecting the earth’s ecosystem, including rodent disturbance inducing functional biodiversity change in a desert ecosystem.

Soil fungal community needs nutrients for growth that drives nutrient cycling by hydrolyzing and immobilizing complex biomolecules and decomposing organic matter (from plant residues, pathogens, etc.) (He et al., 2008; Raaijmakers et al., 2009). Likewise, the highly abundant and widely distributed bacterial community plays an important role in soil material cycling and energy flow, including the mineralization of organic matter and biogeochemical cycling of carbon and nitrogen (Chatterjee et al., 2008; Caporaso et al., 2011). Notably, the bacterial community is susceptible to environmental factors (natural and man-made). Overall, soil microbial communities help maintain soil properties and functions promoting plant growth and development and maintaining the diversity and functional balance of an ecosystem. However, the comprehensive assessment of change in microbial community structure and diversity by gerbil disturbances in desert ecosystems is largely unknown, especially in the rhizosphere (one of the most prominent areas of soil activity and diversity).

*Haloxylon ammodendron* is the most widely distributed vegetation in desert areas. It has vital ecological significance and economic value in preventing dune flow, offering nutrients and water conditions necessary for the survival of other vegetation and animals (vertebrates and invertebrates), and maintaining the function and structure of arid ecosystems (Zhao et al., 2019). Meanwhile, the great gerbil (*Rhombomys opimus*, called gerbil from hereon), the main rodent in the desert and semi-desert areas of northwest China, is a highly social animal. It is good in burrowing, nests in plant roots, and eats from desert plants including *Kalidium foliatum*, *Tamarix taklamakanensis*, *Caragana sinica*, especially the phloem and branches of *H. ammodendron* (Wei et al., 2012; Zhao W. et al., 2021). To ensure their habitat and food source, gerbils form holes in the *H. ammodendron* root system, which worsens the woodland conditions due to the loss of a large amount of space affecting the development of the vegetation root system. This impacts the growth and development of the *H. ammodendron* forest in the Gurbantunggut Desert weakening the desert ecosystem (Davidson and Lightfoot, 2008; Villarreal et al., 2008). However, how the gerbils’ burrowing activities affect the rhizosphere microecology of desert plants, especially the founder species *H. ammodendron*, is not clear. In this context, exploring the effect of gerbil disturbance on the soil microbial community diversity in the rhizosphere of *H. ammodendron* can help understand the soil microecology response to gerbil disturbance in the growth of *H. ammodendron* can help protect/restore the desert ecosystem.

In this study, we quantified gerbil disturbance intensities (no disturbance, mild disturbance, moderate disturbance, and severe disturbance) and used high-throughput sequencing (HTS) to explore the impact of gerbil disturbance on the microbial diversity and structure (fungi and bacteria) at different soil depths (0–20, 20–40, 40–60 cm). We hypothesized that the soil characteristics of *H. ammodendron* rhizosphere at different soil depths changed with the gerbil disturbance intensities, and the composition of microbial community was regulated. The objectives of this study were: (1) to clarify the effects of gerbil disturbance on soil nutrients in desert ecosystems; (2) to investigate the composition and structure of soil bacterial and fungal communities associated with gerbil disturbance; and (3) to reveal the remolding effect of gerbil disturbance on soil microenvironment of *H. ammodendron* rhizosphere in desert ecosystems in northwest China to further understand the potential correlations with changes in the microbial community at a regional scale.

## Materials and methods

### Study sites and field sampling

The research area is located on the southern edge of the Gurbantunggut Desert (44°26′∼45°12′N, 86.2°06′∼87°54′E; altitude 314–436.8 m) in northwest China. It has a typical temperate arid desert climate with a hot summer and cold winter. The annual average temperature and rainfall were 7.1°C and 215.6 mm, respectively. The rainfall was concentrated in summer and winter, accounting for 75% of the annual rainfall; the annual evaporation was >2000 mm (Cui et al., 2017). The soil was gray desert soil. Shrubs plants *Tamarix ramosissima*, *H. ammodendron*, and *Haloxylon persicum* dominated the vegetation with ∼30% coverage; *H. ammodendron* is the main construction species (Huang et al., 2015). Herbaceous layer plants mainly include ephemeral plants (such as *Alyssum linifolium*, *Eremurus inderiensis*, and *Leptaleum filifolium*) and small perennials (such as *Myosotis scorpioides*, *Salicornia brachiate, Erodium oxyrrhynchum*, and *Ceratocarpus arenarius*) cover ∼40% of the study area (Fan et al., 2013).

**FIGURE 1:**
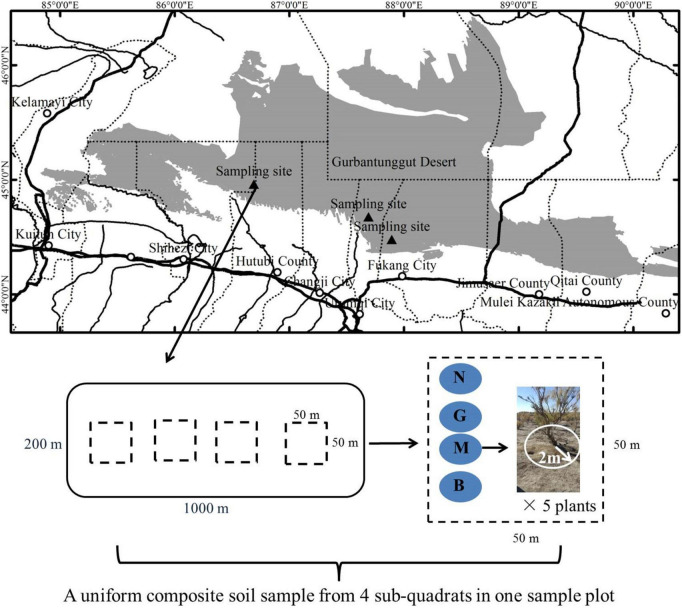
The position of the research area on the southern edge of the Gurbantunggut Desert.

Field investigation and the collection of gerbil disturbed *H. ammodendron* rhizosphere samples were conducted from June to early July 2020 (peak season of *H. ammodendron* growth). Since the structure of the gerbil’s burrow system is relatively complex, it is generally divided into two layers, the upper (20–40 cm above the ground) and lower (∼80 cm above the ground) layers. The gerbil activity is mostly distributed in the upper layer (Li and Abdukadir, 2004). Accordingly, in this study, the sample soil depth was set at 0–20, 20–40, and 40–60 cm layers. *H. ammodendron* located in the center of the gerbil burrow area was taken as the center point, and then an effective burrow circular area with a radius of 2 m was determined based on the stealing opening burrow method (i.e., all the gerbil holes in the quadrat were slightly blocked by sand and soil, and the number of burrows reopened after 48 h were considered real burrows) (Zhao et al., 2007). The degree of gerbil disturbance was quantified according to the number of effective openings under the shrub and its growth in that quadrat. The gerbil disturbance was graded into four intensities: none (N; 0 effective burrows); mild (G; 1–3 effective burrows); moderate (M; 3–6 effective burrows), and severe (B; >6 effective burrows). This study examined 3 sample plots (1000 × 200 m) (Figure 1), and to ensure equal data representation, each plot contained 4 sub-quadrats with different disturbance intensities (50 × 50 m; a total of 12 quadrats in 3 plots). Twelve homogeneous composite samples (3 depth × 4 intensity) of different soil depths were collected from each sample plot, each sub-composite sample represented the rhizosphere of five randomly selected *H. ammodendron* plants with the same degree of disturbance (excavated until found plant roots). After the removal of plant litter and bulk soil, rhizosphere soil samples at different depths (0–20, 20–40, and 40–60 cm) were mixed to obtain uniform composite soil with different disturbance intensities. Each mixed soil sample was divided into 2 parts, one was stored in liquid nitrogen until microbial DNA extraction, and the other was subjected to soil physicochemical analysis. In total, 72 test samples, 36 each for microbiological and physicochemical analyses (4 intensity × 3 depths × 3 replicates) were processed.

### Soil physicochemical analyses

The rhizosphere soil samples were naturally air-dried to constant weight in the laboratory and then passed through a 2 mm sieve before the determination of soil nutrient content. Soil total organic carbon (TOC) was measured by external heating of soil samples with potassium dichromate. Total nitrogen (TN) was measured using the perchloric acid-sulfuric acid digestion method and an automatic nitrogen analyzer (FIAstar™ 5000; FOSS, Gothenburg, Sweden) (Hood-Nowotny et al., 2010). Total potassium (TK), available potassium (AK), and total salt (TS) contents were analyzed using atomic absorption spectrometry (Bao, 2008). Total phosphorus (TP) and available phosphorus (AP) contents were determined by acid digestion (H_2_SO_4_ + HClO_4_) and sodium bicarbonate extraction methods, respectively, using the molybdenum blue colorimetric method (Hao et al., 2018). Available ammonia (AN; NH_4_-N) content was estimated by 0.01 M calcium chloride extraction and a flow analyzer (AutoAnalyzer 3, Germany).

### DNA extraction

Total genome DNA was extracted from fully mixed soil samples (0.5 g each) using the FastDNA^®^ Spin Kit for Soil (MP Biomedicals, Santa Ana, CA, United States) following the manufacturer’s protocol. DNA concentration and purity were detected by NanoDrop2000 (Thermo Fisher Scientific, Waltham, MA, United States), and DNA integrity was monitored by 1% agar-gel electrophoresis. Extracted DNA samples were diluted to 1 ng/μL with sterile water and then used as a template for PCR.

### Amplification of 16S rRNA and internal transcribed spacer genes from soil materials

Three replicates of polymerase chain reaction (PCR) were set up for each sample using genomic DNA as the template and specific primers. Primers for the amplification of 16S rRNA gene V4 region (515 F: 5′-GTGCCAGCMGCCGCGGTAA-3′; 806 R: 5′-GGACTACHVGGGTWTCTAAT-3′) and internal transcribed spacer (ITS) gene 5F region (1737 F: 5′-GGAAGTAAAAGTCGTAACAAGG-3′; 2043 R: 5′-GCTGCGTTCTTCATCGATGC-3′) were with specific barcodes (Zhang et al., 2016; Guo et al., 2017). All PCRs were carried out with highly efficient and accurate Phusion^®^ High-Fidelity PCR Master Mix (New England Biolabs, Ipswich, MA, United States) in a thermocycler (ABI GeneAmp^®^9700, ABI, United States). The PCR program for the 16S rRNA gene V4 region was as follows: 95°C for 3 min (initial denaturation), followed by 30 cycles of 95°C for 30 s, 55°C for 30 s, 72°C for 30 s, and a final extension step at 72°C for 5 min. The PCR program for ITS rRNA of fungi was as follows: 95°C for 3 min, followed by 32 cycles of 95°C for 30 s, 52°C for 30 s, 72°C for 30 s, and a final extension step at 72°C for 5 min.

### Purification and sequencing of polymerase chain reaction products

The PCR products, mixed with 1 × loading buffer (contained SYB green), were resolved by 2% agarose gel electrophoresis. Target strips (16S V4 region ∼400 bp, ITS1 ∼300 bp) were cut and purified using the GeneJET™ Gel Extraction Kit (Thermo Scientific) following the manufacturer’s instructions and isolated DNA was quantified using the Quantus™ Fluorometer (Promega, Madison, WI, United States). The Ion Plus Fragment Library Kit 48 rxns (Thermo Scientific) was used to generate the sequencing libraries and their quality was evaluated using Qubit^®^2.0 fluorometer (Thermo Scientific) and Agilent Bioanalyzer 2100 system. Finally, the library was sequenced on the Illumina HiSeq2500 platform and 400/600 bp single-end reads were generated.

### Bioinformatics and statistical analysis

Based on unique barcodes, single-end reads were assigned to respective samples using Cutadapt^1^ (V1.9.1) (Martin, 2011) and truncated to remove barcode and primer sequences. The remaining paired-end reads of each sample were spliced using FLASH^2^ (V1.2.7) (Magoč and Salzberg, 2011) and then assembled to generate raw tags. Quality filtering of the raw reads was strictly and accurately performed under specific filtering conditions to obtain high-quality clean reads using Cutadapt (V1.9.1, see text footnote 1) quality-control process (Bokulich et al., 2013). The reads were compared with the reference database (Unite database^3^) (Alawneh et al., 2006) using the UCHIME algorithm (UCHIME Algorithm^4^) (Edgar et al., 2011) to find and remove chimera sequences. Lastly, the Clean Reads were obtained.

Microbial communities analysis was carried out by Uparse software (Uparse v7.0.1001^5^) (Edgar, 2013). Sequences with ≥97% similarity were assigned to the same operational taxonomic units (OTUs). A representative sequence (highest frequency) for each OTU was screened for further annotation.

Each representative sequence was blasted against the Unite (for ITS region; see text footnote 3) or Silva databases (for 16S rRNA gene region^6^) using the QIIME software^7^ (Version 1.9.1) and annotated for taxonomic information. Multiple sequence alignment was performed with the MUSCLE software^8^ (Version 3.8.31) (Edgar, 2004) to find phylogenetic relationships among different OTUs and the dominant species in different samples. OTUs abundance was normalized using a standard sequence number corresponding to the sample with the least sequences. Subsequently, alpha and beta diversity analyses were performed for this normalized data. The raw reads have been deposited into the NCBI Sequence Read Archive (SRA) database; the BioProject accession numbers for 16S rRNA gene and ITS region sequences are PRJNA787100 and PRJNA787528, respectively.

Alpha diversity analyses include four representative indices, Chao1, Shannon, Simpson, and ACE. These are employed to analyze the species diversity of the microbial community in a sample. Among them, Chao 1 and ACE estimators show the community richness, while Shannon and Simpson’s indices reflect the community diversity. Alpha diversity analyses were performed with QIIME (Version 1.7.0) and displayed with R software (Version 2.15.3).

Beta diversity analysis was used to evaluate the microbial species diversity between the two samples. Beta diversity on weighted unifrac was performed by QIIME software (Version 1.7.0). Principal Coordinate Analysis (PCoA) was performed to get principal coordinates and visualize the complex, multidimensional data, that was displayed by the WGCNA, stat, and ggplot2 packages in R software (Version 2.15.3). Before PCA, data were subjected to cluster analysis to reduce the dimension of the original variables using the FactoMineR and ggplot2 packages in R software. In addition, R software was used to perform the Wilcoxon rank-sum test, Spearman correlation analysis of heat maps (pheatmap package), and canonical correlation analysis (CCA) (cca analysis in vegan package). LEfSe (LDA Effect Size) analysis using the LEfSe software (the default screening value of LDA Score was 4) was used to find biomarkers with statistical differences between groups.

The statistical analysis of variance was performed with SPSS 19.0 (IBM Inc., Armonk, NY, United States) followed by Bonferroni’s statistical test for multiple comparisons; the significance level cut-off was 0.05. To evaluate the correlation between diversity index and soil nutrient content, stepwise multiple linear regression model analysis was performed by SPSS version 19.0 and visualized by GraphPad Prism 5 software. The contribution of individual abiotic factors (soil nutrient, soil salinity, degree of gerbil disturbance, and soil depth) to fungal community structure was studied by the structural equation model (SEM) through the AMOS 24.0 software. Shannon index and ACE estimator were used as the structural indicators of the fungal community.

## Results

### Gerbil disturbance affected the soil nutrient contents

The results showed that gerbil disturbance significantly impacted the most soil nutrient contents (*p* < 0.05). Mild (G), moderate (M), and severe (B) disturbances significantly raised the total phosphorus (TP) and ammonium nitrogen (AN) contents compared to no disturbance (N) (*p* < 0.05) (Table 1). Conversely, G, M, and B significantly decreased the total salt (TS) content compared with N (*p* < 0.05) (Table 1). Furthermore, soil available phosphorus (AP) content was dramatically lower for M compared with N, G, and B. Available potassium (AK) content was markedly higher for G than for M (Table 1) (*p* < 0.05). In terms of soil depth, soil organic carbon (TOC), soil total nitrogen (TN), and AK contents were significantly higher in the 0–20 cm layer than in the 20–40 and 40–60 cm layers (*p* < 0.05), while the other soil properties [TP, soil total potassium (TK), AN, AP, and TS] were not significantly different (*p* > 0.05) (Table 1).

**TABLE 1:** Effect of gerbil disturbance and soil depths on the soil properties.

Variables	TOC (g/kg)	TN (g/kg)	TP (g/kg)	TK (g/kg)	AN (mg/kg)	AP (mg/kg)	AK (mg/kg)	TS (mg/kg)
N	1.346 ± 0.130 a	0.057 ± 0.007 a	0.417 ± 0.005 b	22.789 ± 0.727 a	19.926 ± 1.897 b	4.790 ± 0.137 b	418.444 ± 52.380 ab	1.818 ± 0.093 a
G	1.654 ± 0.086 a	0.076 ± 0.010 a	0.439 ± 0.007 b	23.611 ± 0.613 a	35.798 ± 1.343 a	4.622 ± 0.392 b	501.167 ± 29.259 a	0.927 ± 0.036 b
M	1.667 ± 0.148 a	0.101 ± 0.021 a	0.436 ± 0.012 b	22.706 ± 0.549 a	33.648 ± 1.329 a	2.777 ± 0.160 a	359.889 ± 0.702 b	0.813 ± 0.074 b
B	1.579 ± 0.245 a	0.075 ± 0.012 a	0.479 ± 0.009 a	24.338 ± 0.648 a	39.869 ± 2.344 a	4.865 ± 0.303 b	491.333 ± 22.308 ab	0.916 ± 0.076 b
0–20 cm	2.126 ± 0.099 a	0.121 ± 0.013 a	0.443 ± 0.006 a	22.909 ± 0.480 a	34.915 ± 1.624 a	4.713 ± 0.304 a	540.292 ± 22.588 a	0.929 ± 0.124 a
20–40 cm	1.243 ± 0.079 b	0.049 ± 0.004 b	0.443 ± 0.011 a	23.529 ± 0.754 a	33.046 ± 3.691 a	4.408 ± 0.408 a	439.125 ± 30.651 b	1.240 ± 0.142 a
40–60 cm	1.316 ± 0.051 b	0.062 ± 0.004 b	0.442 ± 0.012 a	23.645 ± 0.426 a	28.970 ± 2.067 a	3.669 ± 0.222 a	348.708 ± 23.050 b	1.186 ± 0.126 a

N, G, M, and B represent the gerbil none, mild, moderate, or severe disturbances, respectively. 0–20, 20–40, and 40–60 cm represent different soil depths. Value is Mean ± standard error, different lower-case letters represented a significant difference (p < 0.05) was assessed by one-way analysis of variance followed by Bonferroni’s statistic test for multiple comparisons, the same letter indicates no significant difference (p > 0.05). Abbreviations: TOC, soil organic carbon; TN, soil total nitrogen; TP, soil total phosphorus; TK, soil total potassium; AN, soil available ammonia; AP, soil available phosphorus; AK, soil available potassium; TS, soil total salt.

### Gerbil disturbance affected the soil microbial diversity and structure

Alpha diversity analyses revealed significant differences in the bacterial community across different intensities of gerbil disturbance (*p* < 0.05) (Supplementary Figures 1A,C). The Shannon and Chao1 indices significantly increased from N to M (*p* < 0.05), with a further mild increase in the B group. Surprisingly, the alpha diversity indices showed no significant differences among soil fungal communities for different intensities of gerbil disturbance (*p* > 0.05) (Supplementary Figures 1B,D). It suggests that gerbil disturbance only affected the diversity (Shannon index) and richness (Chao 1 index) of soil bacterial community in the arid desert system.

**FIGURE 2:**
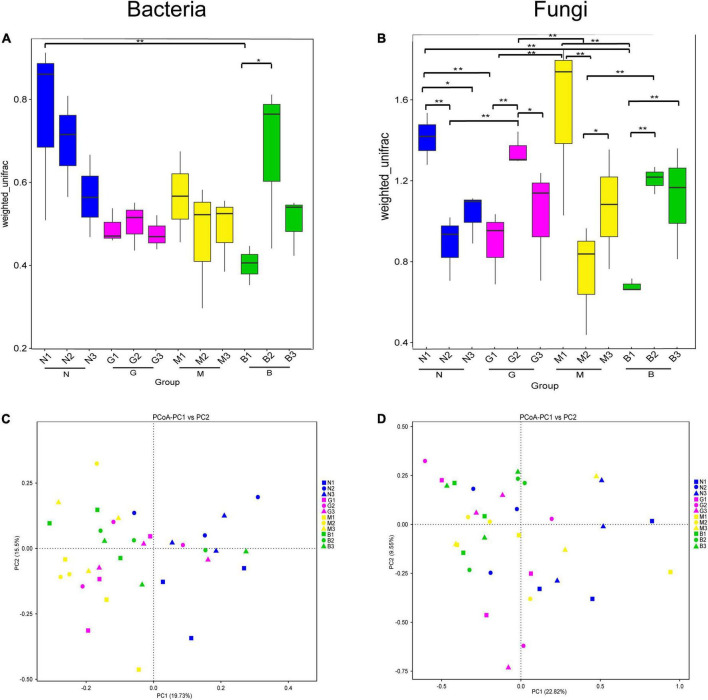
Beta diversity analysis of soil microbial community based on weighted UniFrac distance. **(A,B)** The significance test of the differences of Beta diversity, which the * represented a significant difference (*p* < 0.05) assessed by Wilcoxon rank-sum test for analysis. Ordinate is the Beta diversity; abscissa is the group name (N, G, M, and B represent the gerbil none, mild, moderate, or severe disturbances, respectively; 1, 2, and 3: soil depth 0–20, 20–40, and 40–60 cm, respectively). For example, N1 represents at a soil depth of 0–20 cm with gerbil none disturbance sample. **(C,D)** Principal Co-ordinates Analysis (PCoA), which each point in the diagram represents a sample, and samples from the same group are represented in the same color. ** represents significances at *p* < 0 .01.

Next, beta diversity analyses showed a significant difference in the microbial communities based on the Wilcox rank-sum test for different intensities of gerbil disturbance (Figure 2). As shown in Figure 2A, N1 and B2 samples had significantly higher bacterial diversity than the B1 sample. This indicated that B significantly affected the bacterial community beta diversity in the 0–20 cm soil layer. Likewise, the soil fungal community showed higher diversity in the N1 sample than in the N2, N3, B1, and G1 samples; the G2 sample was significantly higher than the G1, G3, N2, and M2 samples. M1 sample was significantly higher than the G1, M2, and B1 samples. B2 and B3 samples were both significantly higher than the B1 sample. B2 and M3 samples were both significantly higher than the M2 sample (Figure 2B). These results indicated that gerbil disturbance significantly and differently affected the structure of soil microbial communities at different soil depths. The results of the beta diversity analysis were confirmed by PCoA based on weighted UniFrac distances. As shown in Figure 2C, for the soil bacterial community, samples showed good separation from each other according to PC1 (19.73%) and PC2 (15.5%). Likewise, for the soil fungal community too, samples were separated from each other according to PC1 (22.82%) and PC2 (9.55%) (Figure 2D). This indicated that gerbil disturbance and soil depth significantly affected the similarity of soil microbial community structure.

### Gerbil disturbance affected soil microbial composition

**FIGURE 3:**
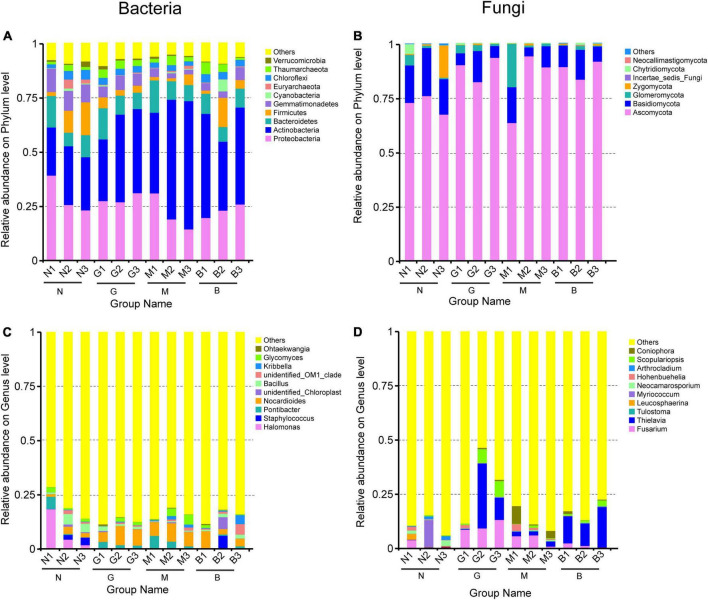
Histograms of relative abundance of the top 10 soil bacterial community **(A,C)** and soil fungi community **(B,D)**. Ordinate is the relative abundance of species; others mean less or not annotated; abscissa is the group name (N, G, M, and B represent the gerbil none, mild, moderate, or severe disturbances, respectively; 1, 2, and 3: soil depth 0–20, 20–40, and 40–60 cm, respectively). For example, N1 represents at a soil depth of 0–20 cm with gerbil none disturbance sample.

At the phylum level, the bacterial community was overwhelmingly dominated by Proteobacteria, Bacteroidetes, Actinobacteria, Gemmatimonadetes, and Firmicutes (Figure 3A) in gerbil disturbed samples. Increased intensity of disturbance increased the relative abundance of Actinobacteria, but greatly declined the relative abundance of Proteobacteria, Gemmatimonadetes, and Firmicutes compared with the N samples (Figure 3A). Furthermore, increasing soil depth showed a relatively low abundance of Proteobacteria and Gemmatimonadetes in the N and M samples but these were significantly higher in the B sample. The relative abundance of Bacteroidetes in the G and M samples showed a significant reduction with the increase in soil depth. At the genus level, *Nocardioides* were more abundant than other genera, with the relative abundance ranging from 1.214 (in N1) to 8.824% (in G2). Increasing intensity of gerbil disturbance increased the relative abundance of *Nocardioides*, but greatly declined the relative abundance of *Halomonas* compared with N (Figure 3C). Furthermore, with soil depth increase significantly reduced the relative abundance of *Halomonas* in N samples and *Pontibacter* in N, G, M samples but significantly increased the relative abundance of *Staphylococcus* in N, M samples. In conclusion, gerbil disturbance at different soil depths significantly altered the composition of the soil bacterial community.

Likewise, soil fungal community analysis revealed that Ascomycota was the most dominant phylum with a relative abundance of 63.274 (in M1) to 93.359% (in G3) (Figure 3B). Increasing intensity of gerbil disturbance increased the relative abundance of Ascomycota, *Thielavia*, *Tulostoma*, *Scopulariopsis*, and *Coniophora*, but greatly declined the relative abundance of Basidiomycota, Zygomycota, *Leucosphaerina*, *Myriococcum*, *Neocamarosporium*, and *Arthrocladium* compared with N (Figures 3B,D). Furthermore, an increase in soil depth significantly reduced the relative abundance of Glomeromycota in N and G samples.

**FIGURE 4:**
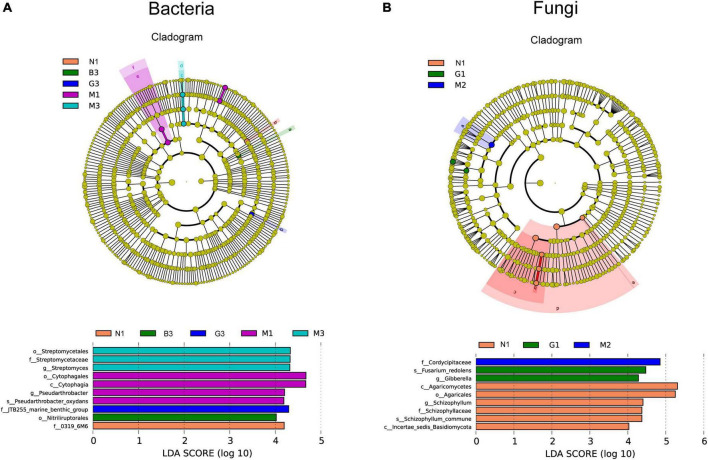
Linear discriminant analysis effect size (Lefse) analysis of differences in soil microbial community [bacterial **(A)** and fungi **(B**)] composition as a function of gerbil disturbance intensity. In cladograms, the circle radiating from inside to outside represents the taxonomic level from the Phylum to the species. Each small circle at a different taxonomic level represents microbial groups that were significantly enriched in the corresponding groups and that significantly influenced the differences between groups, and the diameter of the small circle is proportionate to the relative abundance of species. Light yellow small circle represents microbial groups with no significant differences. The length of the histogram represents the size of the influence of species with significant differences. Group name (N, G, M, and B represent the gerbil none, mild, moderate, or severe disturbances, respectively; 1, 2, and 3: soil depth 0–20, 20–40, and 40–60 cm, respectively). For example, N1 represents at a soil depth of 0–20 cm with gerbil none disturbance sample.

Linear discriminant (LDA) and Effect Size (LEFSe) analyses were performed to explore the statistically significant differences in the abundance of soil microbial communities and find the biological relevance of these differences concerning gerbil disturbance. As shown in Figure 4, 10 biomarkers within the soil bacterial community with significant differences in abundance were found (Figure 4A) including 1 in N1, 1 in B3, 1 in G3, 4 in M1, and 3 in M3 samples. Among these, *Streptomyces* (in M3) and *Pseudarthrobacter* (in M1) represent the genera. Likewise, 9 biomarkers within the soil fungal community with significant differences in abundance were found including 6 in N1 (Agaricomycetes, Agaricales, *Schizophylum*, Schizophyllaceae *Schizophyllum*_*commune*, and Incertae_sedis_Basidiomycota), 2 in G1 (*Fusarium*_*redolens* and *Gibberella)*, and 1 in M2 (Cordycipitaceae) samples (Figure 4B).

### Relationships between soil microbial community structure and soil properties

Spearman’s heat map revealed the correlation between the top 35 bacterial OTUs and soil nutrient content. As shown in Supplementary Figure 2A, TOC, TP, TK, and AP contents showed a significantly negative correlation with OTU-38 (*R*^2^ < 0, *p* < 0.05), which belongs to the genus *Nocardioides*, phylum Actinobacteria. TOC, TN, TP, and AP contents showed a significantly negative correlation with OTU-60 (*R*^2^ < 0, *p* < 0.05), which belongs to the genus *Fusobacterium*, phylum Fusobacteria. TOC, TP, AK, and AP contents showed a significant negative correlation with OTU-16 (*R*^2^ < 0, *p* < 0.05), which belongs to the genus *Kribbella*, phylum Actinobacteria. TOC, TN, TP, and AP contents showed a significantly positive correlation with OTU-33 (*R*^2^ > 0, *p* < 0.05), which belongs to the genus *Pontibacter*, phylum Bacteroidetes.

Likewise, Spearman’s heat map revealed the correlation between the top 35 fungal OTUs and soil nutrient content. OTU-7 and OTU-4 showed a significantly positive correlation with AP, and TP contents (*R*^2^ > 0, *p* < 0.05) (Supplementary Figure 2B). AP content positively correlated with OTU-2, OTU-8, OTU-1914, OTU-48, OTU-14, and OTU-32 (*R*^2^ > 0, *p* < 0.05) but showed significantly negative correlation with OTU-51, OTU-837, OTU-96, OTU-27, OTU-24, OTU-35, OTU-25, OTU-699, OTU-38, and OTU-34 (*R*^2^ < 0, *p* < 0.05). TN, AN, and AK contents significantly positively correlated with OTU-994 (*R*^2^ > 0, *p* < 0.05). OTU-7, OTU-4, OTU-2, OTU-1914, OTU-27, OTU-14, and OTU-24 belong to the phylum Ascomycota. OTU-8, OTU-837, OTU-96, OTU-25, OTU-699, OTU-38, and OTU-994 belong to the genera *Leucosphaerina, Fusarium, Neocamarosporium, Engyodontium, Retroconis, Scopulariopsis*, and *Thielavia*, respectively, corresponding to the phylum Ascomycota. OTU-48, OTU-32, and OTU-51 correspond to the genera *Myriococcum*, *Tylospora*, and *Tulostoma*, respectively, belonging to the phylum Basidiomycota.

**FIGURE 5:**
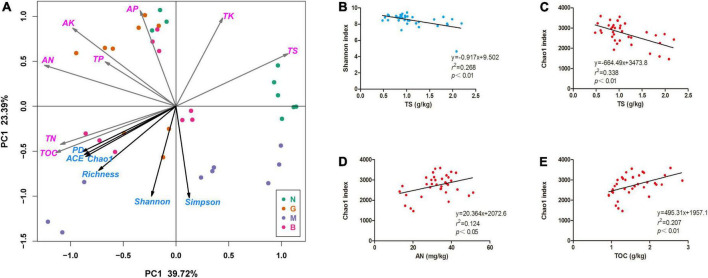
Relationship between the Alpha diversities indices of soil bacterial community and soil physicochemical characteristics. **(A)** Principal component analysis (PCA) based on soil physicochemical characteristics as variables, and Alpha diversities indices of soil bacteria community were fitted as factors with significance < 0.05 onto the Ordination. **(B–E)** Linear regression relationships between soil nutrition content and Alpha diversity indices (Shannon index and Chao 1 index) of soil bacterial community. N, G, M, and B represent the gerbil none, mild, moderate, or severe disturbances, respectively. Abbreviations: TOC, soil organic carbon; TN, soil total nitrogen; TP, soil total phosphorus; TK, soil total potassium; AN, soil available ammonia; AP, soil available phosphorus; AK, soil available potassium; TS, soil total salt.

**FIGURE 6:**
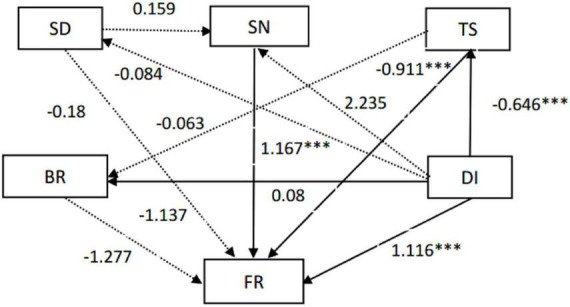
Structural equation model (SEM) between environmental factors and soil fungal community. The solid line indicates that the influence is significant (*p* < 0.05), and the dotted line indicates that the influence is not significant (*p* > 0.05). Abbreviations: SD, soil depth; SN, soil nutrition content; TS, soil total salt; DI, gerbil disturbance; BR, soil bacteria community structure; FR, soil fungi community structure. ***represents significances at *p* < 0 .001.

The PCoA of soil factors revealed that soil samples N, G, M, and B clustered together in relative isolation, indicating large environmental heterogeneity in these soil samples (Figure 5A). Alpha diversity indices including Simpson, Shannon, Phylogenetic diversity (PD), ACE, Chao1, and observed species (Richness) fitting onto the Ordination revealed that these positively correlated with soil TOC and AN content, but negatively correlated with TS content (Figure 5A). This was consistent with the output of the stepwise multiple linear regression model between alpha diversity of soil bacterial communities and soil factors (Figures 5B–E). Notably, Spearman’s correlation test showed that soil factors had no significant effect on the alpha diversity of soil fungal community, but significantly affected the alpha diversity of soil bacterial community (Supplementary Table 1) in gerbils disturbed soils. In addition, the structural equation model (SEM) showed that the difference in soil fungal community structure was due to the direct reverse effect of TS content and the indirect positive effect of soil nutrients and gerbil disturbance (Figure 6).

**FIGURE 7:**
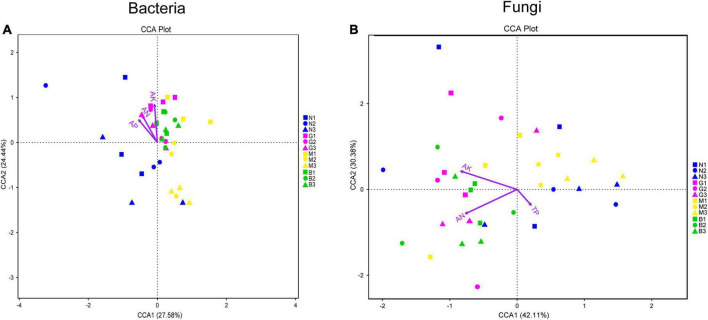
Relationship between soil physicochemical characteristics and soil microbial community [bacterial **(A)** and fungi **(B)**]. Canonical Correlation analysis (CCA) based on OTU levels that mainly used to reflect the relationship between soil microbial community and soil physicochemical characteristics. Environmental factors are generally represented by arrows. The length of the arrow line represents the degree of correlation between a certain environmental factor and community and species distribution, and the longer the arrow, the greater the correlation. When the angle between the environmental factors is acute, it means that there is a positive correlation between the two environmental factors, while when the angle is obtuse, there is a negative correlation. Abbreviations: AN, soil available ammonia; AP, soil available phosphorus; AK, soil available potassium; TP, soil total phosphorus. Group name (N, G, M, and B represent the gerbil none, mild, moderate, or severe disturbances, respectively; 1, 2, and 3: soil depth 0–20, 20–40, and 40–60 cm, respectively). For example, N1 represents at a soil depth of 0–20 cm with gerbil none disturbance sample.

Furthermore, canonical correlation analysis (CCA) showing the effects of soil nutrient content on the composition of the microbial community is shown in Figure 7. As shown in Figure 7A, soil AN (*r*^2^ = 0.419, *p* < 0.01), AP (*r*^2^ = 0.440, *p* < 0.01), and AK (*r*^2^ = 0.582, *p* < 0.01) contents were the key driving factors affecting the distribution of soil bacterial community accounting for 52.02% of the explained variation by CCA1 and CCA2 (Figure 7A and Supplementary Table 2). In terms of soil fungal communities, the first ordination axis showed a significant association with soil AN (*r*^2^ = 0.772; *p* < 0.01) and AK (*r*^2^ = 0.757; *p* < 0.01) contents contributing 42.11% of the total variability. The second ordination axis significantly correlated with TP (*r*^2^ = 0.145; *p* < 0.05) with 30.38% contribution rate (Figure 7B and Supplementary Table 2).

## Discussion

The effect of rodent disturbance, through contributing to change in soil factors, on rhizosphere soil microbial community of dominant construction species *H. ammodendron* in the desert ecosystem is an important ecology subject. However, it is a complex phenomenon that is very difficult to quantify. Here, we quantified the intensity of gerbil disturbance at various soil depths to analyze its impact on the soil microbial community structure of *H. ammodendron* rhizosphere. This information provides a strong scientific reference for the sustainable development of the desert ecosystem.

### Gerbil disturbance altered the soil microbial community structure and composition

The soil microbial community can quickly adapt to environmental changes including animal disturbances. Many studies (Jones et al., 1994; Kuznetsova et al., 2013) indicate that rodents, considered ecological engineers, can directly or indirectly affect the structure of soil microbial community in a cave environment. This study found that the microbial alpha diversity of *H. ammodendron* rhizosphere soil in the Gurbantunggut desert showed significant changes under different intensities of gerbil disturbance; the alpha diversity of the bacterial community increased but the fungal community showed the opposite trend (Supplementary Figure 1). This indicated that gerbil disturbance had different effects on soil bacterial and fungal communities. Gao et al. (2021) reported a similar finding that grassland restoration significantly changed the bacterial diversity without affecting the fungal diversity. Notably, gerbil disturbance can break the interaction between soil microorganisms (bacteria-fungi, bacteria-bacteria, algae-fungi) in the desert ecosystem, including competition, symbiosis, antagonism, parasitism, predation, and so on. Furthermore, animal activities including foraging, burrowing, and excretion can regulate microbial diversity in soil ecosystems by altering the competitive interactions between dominant and releasing/inhibiting subordinate microorganisms (Eldridge et al., 2017).

This study used high-throughput sequencing to identify the change in composition and diversity of soil microbial communities in the studied desert ecosystems that strongly correlated to gerbil disturbance at different soil depths in *H. ammodendron* rhizosphere soil. Gerbils’ interference activities such as digging, burying, running, scraping, soil surface biological crust degradation, etc. may reduce the stability of soil surface changing the structure of soil microbial community. This study found that B significantly reduced the beta diversity of the bacterial community in the 0–20 cm soil layer (Figure 2A). Also, gerbil burrowing can move nutrient-poor underground soil to the soil surface reducing soil carbon and nitrogen content (the main substrate and energy source for microbial survival) (Inouye et al., 1987), which inhibits microbial activity (Gallardo and Schlesinger, 1992). Meanwhile, the activity and diversity of underground microorganisms increases due to the accumulation of gerbil urine and feces (Zhong et al., 2010). This may increase the soil nitrogen content, and regulate the activity of soil nitrogenase by improving the abundance of Azotobacter (Kuznetsova et al., 2013).

Soil microbial communities play a key role in soil functions and ecosystem processes, especially in organic decomposition, material cycling, and energy flow (Levy-Booth et al., 2014; Bahram et al., 2018). We found that *H. ammodendron* rhizosphere soil clearly selected specific microbiome including bacterial (Actinobacteria, Proteobacteria Gemmatimonadetes, Firmicutes, *Nocardioides, Halomonas*) and fungal (Ascomycota, Basidiomycota, Zygomycota, Glomeromycota, *Thielavia*, *Tulostoma*, *Scopulariopsis*) communities (Figure 3). Given the universal existence of these microbial taxa in our samples, we assume that these species are closely associated with the growth of *H. ammodendron*, this may mean further studies exploring the response of advantages soil microorganisms to gerbil disturbance may provide important insights into soil microbiology, contributing to the steady development of desert ecosystem.

In the collected samples, the relative abundance of soil bacterial communities (Actinobacteria, Gemmatimonadetes, Firmicutes, Proteobacteria, Bacteroidetes, *Nocardioide*, *Halomonas*, and *Staphylococcus*) at different soil depths was significantly altered by gerbil disturbance (Figures 3A,C). This suggests that some bacterial species may preferentially multiply in certain ecological regions playing specific ecological roles than other microorganisms. Studies (Fierer et al., 2007; Goldfarb et al., 2011) showed that as eutrophic microorganisms, the relative abundance of Proteobacteria and Bacteroidetes increased rapidly when the soil organic carbon content increased. Here, we found that gerbil moderate disturbance (M) promoted Proteobacteria and Bacteroidetes in the shallow layer (0–20 cm) of *H. ammodendron* rhizosphere, indicating that gerbil moderate disturbance was more conducive to promoting the migration of eutrophic microorganisms from deep soil (40–60 cm) to shallow soil (0–20 cm), which would conducive to promoting the material circulation of *H. ammodendron* rhizosphere soil in desert areas.

Ascomycota and Basidiomycota are widely present in many ecosystems (Chang et al., 2021), including farmland and marine ecosystems (Picard, 2017). These play an important role in material cycling. This study showed that even in the extremely harsh ecological environment of the Gurbantunggut Desert, Ascomycota, and Basidiomycota were still the main dominant fungal phyla (Figure 3B). Moreover, we found that gerbil disturbance significantly increased the relative abundance of Ascomycota which may promote the decomposition of soil organic matter (Hugoni et al., 2018). Gerbil activity can transport litter from the aboveground portion to the *H. ammodendron* rhizosphere. This may increase the leaching of dissolved organic compounds promoting microbial activity, which can then accelerate the mineralization of soil nutrients, including nitrogen, phosphorus and potassium, through enzyme metabolism in soil (Joly et al., 2016). Basidiomycota plays an important role in the carbon cycle in contemporary ecosystems. Basidiomycota and its related taxa have a profound economic impact on agriculture, especially forestry, as partners of saprophytes, plant pathogens, and various symbionts, including ectomycorrhizas (Hibbett and Thorn, 2001). The gerbil disturbance significantly reduced the relative abundance of Basidiomycota (Figure 3B), which reflects the health status of the *H. ammodendron* root system in the desert ecosystem. This information can provide some guidance for the research on *H. ammodendron* protection. Certain soil fungi such as *Glomus* (phylum Glomeromycota) reproduce through the mycelium, mycorrhizal spores, or fragments and therefore are more resistant and resilient to ecological disturbances (Daniell et al., 2001), which often play an important role in ecological functions, such as mediating interactions among AMF species. This study found that the relative abundance of Glomeromycota was relatively high under M and G, especially in the 0–20 cm soil layer (Figure 3B). Overall, gerbil disturbance improved surface soil quality and promoted the colonization of beneficial fungi in the rhizosphere of *H. ammodendron*, and increased the shrub resistance to salinity and drought in the desert ecosystem.

According to LEfSe analysis, the gerbil disturbance caused the emergence of several biomarkers of dominant soil bacteria (i.e., Streptomycetales, Streptomycetaceae, Cytophagales, Cytophagia, *Pseudarthrobacter_oxydans*, JTB255_marine_benthic group, *Streptomyces*, Ntriliruptorale, 0319_6M6, *Pseudarthrobacter*) and soil fungi (i.e., Agaricomycetes, Agaricales, *Schizophylum*, Schizophyllaceae, *Schizophyllum*_*commune*, Incertae_sedis_Basidiomycota, *Fusarium_redolens*, *Gibberella*, and Cordycipitaceae) (Figure 4). Therefore, due to changes in soil microbial community structure, the relative abundances of many soil bacterial and fungal taxa were significantly changed with gerbil disturbance intensity at different soil depths, which may subsequently lead to significant changes in their metabolic functions. Although the mechanisms and processes by which gerbil disturbance regulates the metabolic functions of soil microbial communities remain unclear, this study provides a solid basis for further research.

### Gerbil disturbance and soil factors led to the differential distribution of soil microbial community

Soil nutrient content depletion is the key characteristic of a desert ecosystem. The “fat island” effect (Ayarbe and Kieft, 2000), under the shrub of desert vegetation, is an important nutrient accumulation phenomenon observed under limited soil resources. It is the main survival mechanism of a shrub to promote nutrient utilization and adapt to the barren environment in desert areas (Zhao L. et al., 2021). In this study, different intensities of gerbil disturbance increased the soil TOC, TN, TK, TP, and AN contents in the rhizosphere soil of *H. ammodendron*, especially the contents of TP and AN increased significantly (Table 1). These findings are consistent with Moe and Wegge (2008) showing that the feces of axis deer in grazed grasslands significantly changed the physicochemical properties of soil. We speculate that the burrowing behavior of rodents under the *H. ammodendron* shrub increases the soil accumulation and renewal of litter, animal residue, feces, and urine. This alters the process of soil nutrient retention, transfer, and redistribution promoting the formation of “fat island”. Szeman et al. (2008) reported that animals are the carriers of soil nutrients under desert drought conditions. Especially, the complex internal structure of gerbils’ caves can significantly improve the soil aeration, moisture status, and local microtopography (Russell and MacLean, 2008). These changes are conducive to the microbial decomposition of soil nutrients enriching desert soils (Kuznetsova et al., 2013).

Studies (Wang et al., 2020) have shown that increased soil salinity-induced osmotic and ionic stress can limit nutrient uptake by plants. This is also one of the important factors reducing the *H. ammodendron* population. Interestingly, our study found that gerbil disturbance significantly reduced TS content in the rhizosphere soil of *H. ammodendron*, which is consistent with Xiang et al. (2020). It seems that rodent activities can reduce the “salt island” effect, a phenomenon of higher soil salt content under desert vegetation shrubs due to shrub roots absorbing water and transporting salt (Munns and Tester, 2008; He et al., 2018). In addition, PCoA analysis further showed a significant negative correlation between alpha diversity indices of soil bacterial community and soil TS (Figure 5), which is in line with Li et al. (2014). This might be due to the selective pressure of high soil salinity restricting the growth of salt-tolerant microbes reducing microbial activity or restricting electron receptors on microbes. The fact that soil salinity decreased due to gerbil activity may stimulate the activity and composition of *H. ammodendron* rhizosphere microbes. Therefore, gerbil disturbance helps *H. ammodendron* avoid the risk of soil salinization to a certain extent promoting its growth.

The composition of soil microbial communities is largely driven by certain environmental factors (Stegen et al., 2012). For instance, the availability of soil nutrients is critical for the growth and development of both host plants and rhizosphere microbial communities (Li et al., 2020). In this study, gerbil disturbance altered the TP, AN, AP, AK, and TS contents, which may stimulate the assembly of microbial community of *H. ammodendron* rhizosphere soil. This was further supported by CCA analysis that the soil AN, AP, and AK contents were the important environmental factors driving the distribution of soil bacterial communities; AN, AK, and TP contents were found important for the distribution of soil fungal communities (Figure 7 and Supplementary Table 2). It seems that gerbil disturbance changes the soil nutrient content by the spatial distribution of water and fertilizer in cave areas, which significantly impacts the soil microbial communities (Zheng et al., 2020). Being important for information and material exchange between biological and abiotic environments, soil characteristics and changes in soil microbial community play an important role in soil nutrient acquisition and population maintenance of *H. ammodendron*. Studies (Chen et al., 2021) have shown that in biotrophic soil, nitrogen-fixing bacteria, denitrifying bacteria, and cellulose-decomposing bacteria can directly use simple organic matter for more growth and reproduction. Similarly, certain soil fungi, especially Glomeromycota, can establish a special symbiotic relationship with >80% of land plant roots, which mycelium can bridge plant roots and soil nutrient reserves enhancing the solubility and availability of multiple nutrients, improving soil structure (Berruti et al., 2016).

Dissolved soil organic matter is an important energy substrate for microorganisms. The chemical properties of soil carbon drive the structure and function of the soil microbial community (Ng et al., 2014). Previous studies showed that a rich nutrient environment promotes the growth of soil microorganisms (Jiao et al., 2016). However, our study found that nutrient factors such as TN, TK, TP, and AK did not significantly correlate with the alpha diversity of the bacterial community, while TOC and AN showed a positive correlation (Figure 5 and Supplementary Table 1). Perhaps, gerbil disturbance causes the imbalance of the C: N: P ratio in the cave area, which increases nutrient competition among the microbial community (Margesin et al., 2007). This finding indicated that soil carbon and nitrogen content are the main limiting factors of soil fertility in gerbil disturbance affecting the growth of the host plant.

The differences of microbial taxa in communities under different habitats can provide basic information about microbes’ adaption and response to habitat change (Wang et al., 2019). Different intensities of gerbil disturbance induced direct/indirect effects on soil environmental conditions and microbial community, which was supported by structural equation model (SEM) analysis (Figure 6). Exploring the gerbil disturbance-induced changes in soil nutrients and their effects on microbial community structure can provide a future direction for the utilization of microorganisms to balance the desert ecological system.

## Conclusion

In conclusion, based on the results of gerbil disturbance on soil physicochemical properties and soil microbial community of *H. ammodendron* rhizosphere, we concluded that mild gerbil disturbance (1–3 effective burrows) is the most favorable for the growth and development of *H. ammodendron*, whereas, moderate disturbance (3–6 effective burrows) is the maximum *H. ammodendron* can endure. This study highlights the optimal gerbil disturbance mode maximizing the mutually beneficial relationship between *H. ammodendron* and gerbils, and elucidates the ecological response mechanism of *H. ammodendron* rhizosphere soil and microbial community to the disturbance intensity of gerbils in desert regions. This data can be used for the development of a sustained desert ecosystem.

This study investigated the change in bacterial and fungal community composition and structure in the rhizosphere of *H. ammodendron* at different soil depths due to gerbil disturbances and examined their correlation with soil characteristics in the Gurbantunggut Desert. Gerbil disturbance and soil depth altered the structure and composition of soil microbial communities. Compared to soil fungal communities, the impact was higher on bacterial communities in the *H. ammodendron* rhizosphere soil. The soil nutrient content significantly changed with the increase in gerbil disturbance intensity inducing an overall change in the composition and distribution of the soil microbial community. Furthermore, compared to fungal communities, soil bacterial communities were more sensitive to gerbil disturbance altering the content of soil nutrients. Although further studies are needed to characterize the impact of gerbil disturbance on soil microbial metabolism, this study provides valuable information about rodents-microbial interactions in the desert ecosystem.

## Data availability statement

The original contributions presented in the study are publicly available. This data can be found here: https://www.ncbi.nlm.nih.gov/ accession numbers PRJNA787100 and PRJNA787528.

## Author contributions

WZ and HD conceived and designed the research project. WZ and HD performed field investigation, methodology, data analysis and were major contributors in writing the manuscript. TZ and JD performed field investigation and collected the soil samples. YC and LZ collected the soil samples and modified the manuscript. All authors read and approved the final manuscript.

## Footnotes

1
http://cutadapt.readthedocs.io/en/stable/

2
http://ccb.jhu.edu/software/FLASH/

3
https://unite.ut.ee/

4
http://www.drive5.com/usearch/manual/uchime_algo.html

5
http://drive5.com/uparse/

6
http://www.arb-silva.de/

7
http://qiime.org/scripts/assign_taxonomy.html

8
http://www.drive5.com/muscle/
