# Supplementary material for: Great gerbil burrowing-induced microbial diversity shapes the rhizosphere soil microenvironments of Haloxylon ammodendron in temperate deserts
Source: Front Microbiol. 2022 Aug 10;13:960594. doi: 10.3389/fmicb.2022.960594 (PMC9427191; doi:10.3389/fmicb.2022.960594)
Supplement: Supplementary file 1 [file Data_Sheet_1.docx]

Supplementary Material

# Supplementary Figures and Tables

## Supplementary Figures


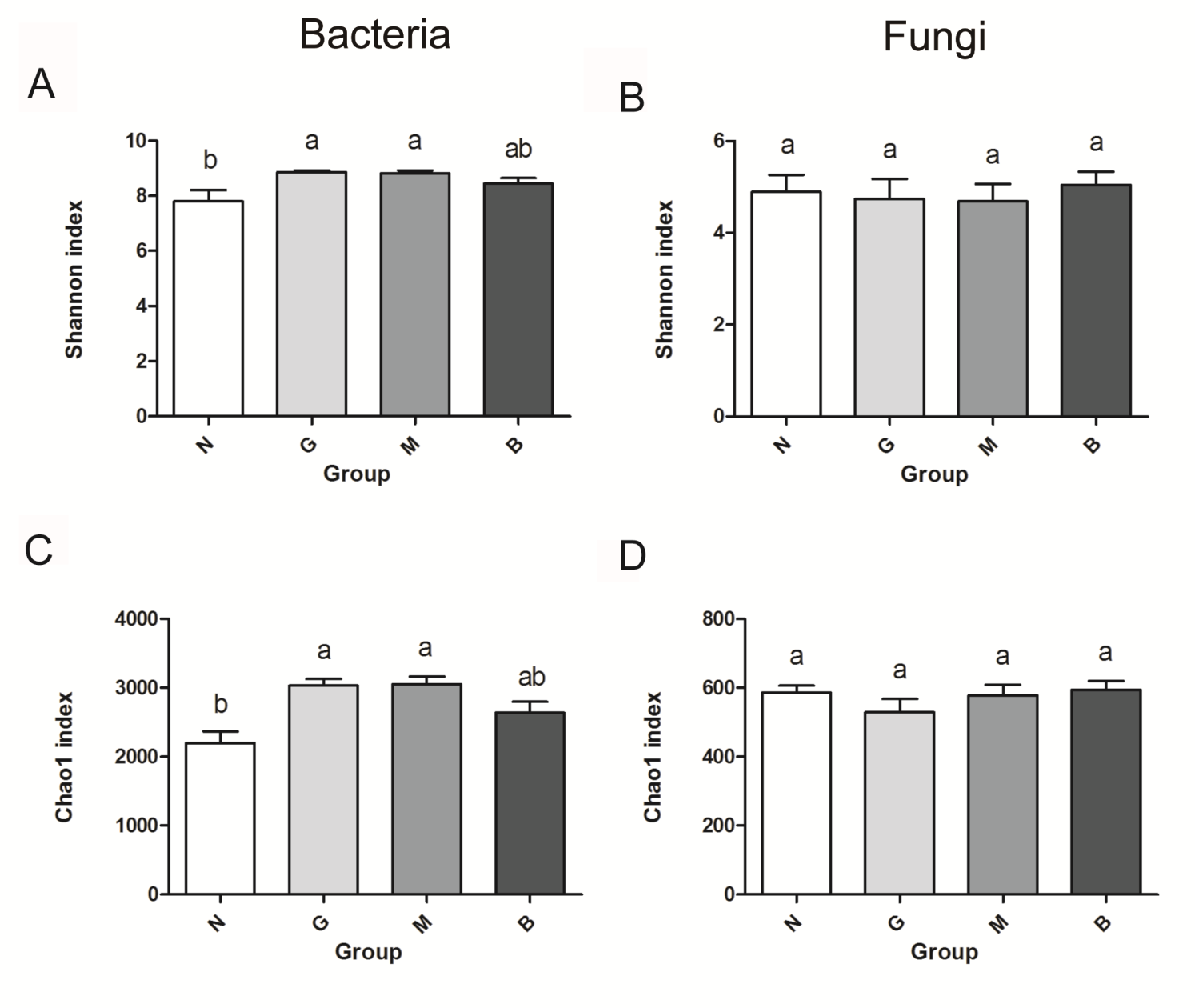


**Supplementary Figure 1.** An analysis of Alpha diversity indices based on Wilcoxon rank-sum test

Description: Bar charts (mean with standard error) with different lower-case letters represented a significant difference (*P* < 0.05) was assessed by one-way analysis of variance followed by Bonferroni's statistic test for multiple comparisons. The same letter indicates no significant difference (*P* > 0.05). Ordinate is Alpha diversity index (Shannon index and Chao 1 index), where (A) (C) represents the bacterial community and (B) (D) represents the fungi community. Abscissa is the group name (N, G, M and B represent the gerbil none, mild, moderate, or severe disturbances, respectively).

**
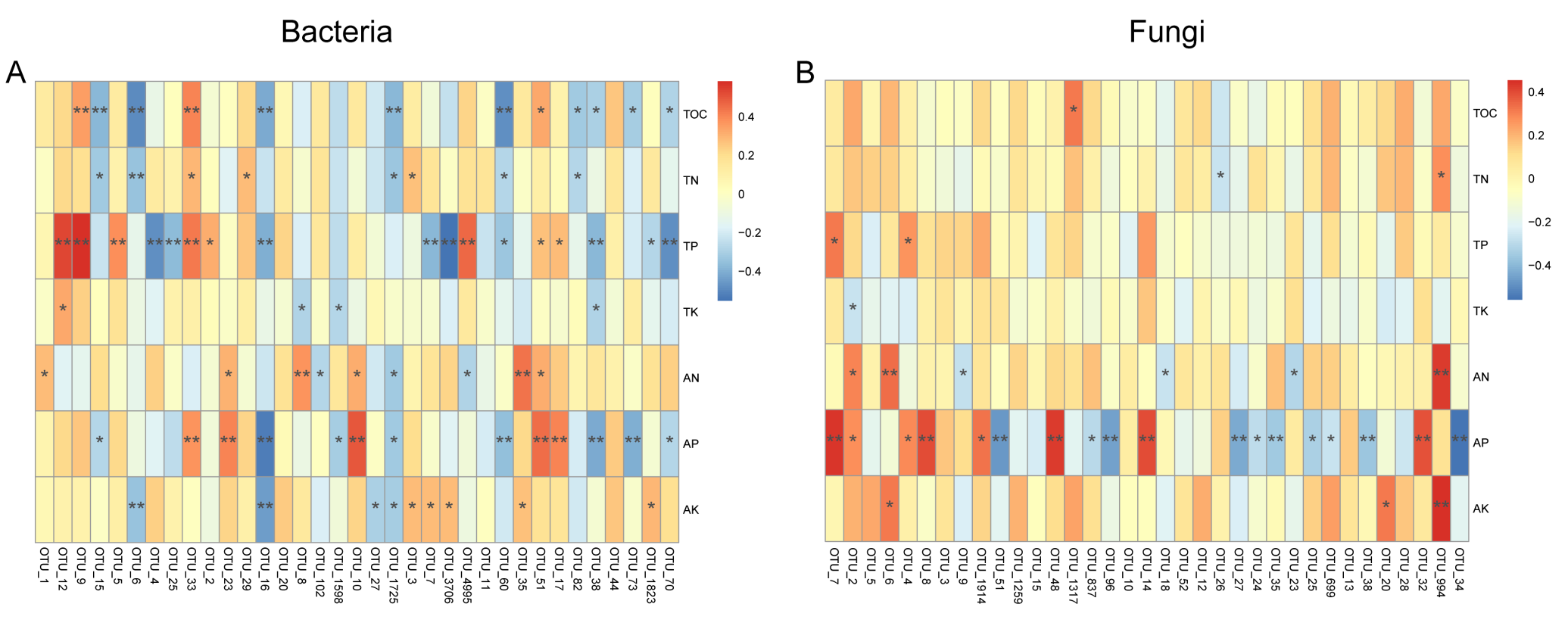
**

**Supplementary Figure 2** Spearman correlation analysis between top 35 OTUs of soil microbial community and soil nutrition content

Description: The mark * is significance test *p <* 0.05. (A) corresponding to bacteria community and (B) corresponding to fungi community. Abbreviations: TOC, soil organic carbon; TN, soil total nitrogen; TP, soil total phosphorus; TK, soil total potassium; AN, soil available ammonia; AP, soil available phosphorus; AK, soil available potassium.

## Supplementary Tables

**Supplementary Table 1** Spearman correlation analysis between alpha diversity of soil microbial community and soil physicochemical properties

| **Variables** | **Bacterial community** | | **Fungal community** | |
| --- | --- | --- | --- | --- |
|  | Shannon | Chao1 | Shannon | Chao1 |
| TOC (g/kg) | 0.229 | **0.455**** | 0.191 | 0.137 |
| TN (g/kg) | 0.189 | 0.291 | 0.126 | 0.105 |
| TP (g/kg) | 0.103 | 0.004 | 0.135 | 0.090 |
| TK (g/kg) | -0.225 | -0.327 | -0.056 | -0.039 |
| AN (mg/kg) | 0.307 | **0.353*** | -0.074 | -0.102 |
| AP (mg/kg) | -0.148 | -0.198 | 0.090 | -0.081 |
| AK (mg/kg) | 0.148 | 0.159 | -0.070 | -0.074 |
| TS (g/kg) | **-0.518**** | **-0.581**** | -0.080 | -0.029 |

Description: the values are the Spearman correlation coefficients. ** means *P*< 0.01; * means *P*< 0.05. Abbreviations: TOC, soil organic carbon; TN, soil total nitrogen; TP, soil total phosphorus; TK, soil total potassium; AN, soil available ammonia; AP, soil available phosphorus; AK, soil available potassium; TS, soil total salt.

**Supplementary Table 2** Results for CCA testing the effects of soil nutrition content on the composition and distribution of soil microbial community

|  | **Bacterial community** | |  | **Fungal community** | |
| --- | --- | --- | --- | --- | --- |
| **Variables** | *r*^2^ | *P* value | **Variables** | *r*^2^ | *P*  value |
| **AN (mg/kg)** | 0.419034713 | 0.00049975 | **TP (g/kg)** | 0.145402766 | 0.019990005 |
| **AP (mg/kg)** | 0.439520629 | 0.00049975 | **AN (mg/kg)** | 0.771910965 | 0.00049975 |
| **AK (mg/kg)** | 0.582057155 | 0.00049975 | **AK (mg/kg)** | 0.757358864 | 0.00049975 |

Description: *r^2^* is the determinant coefficients of the distribution of the soil microbial community by soil factors. *P* value is the *p*-value of significance test, and *P*< 0.05 indicates statistical significance. Abbreviations: AN, soil available ammonia; AP, soil available phosphorus; AK, soil available potassium; TP, soil total phosphorus.
